# Depletion of Cr(VI) from aqueous solution by heat dried biomass of a newly isolated fungus *Arthrinium malaysianum*: A mechanistic approach

**DOI:** 10.1038/s41598-017-10160-0

**Published:** 2017-09-12

**Authors:** Rajib Majumder, Lubna Sheikh, Animesh Naskar, Vineeta, Manabendra Mukherjee, Sucheta Tripathy

**Affiliations:** 1 Structural Biology & Bio-Informatics Division, CSIR-Indian Institute of Chemical Biology, Kolkata, 700032 India; 2 Department of Food Technology and Biochemical Engineering, Jadavpur University, Kolkata, 700032 India; 3 Surface Physics and Material Science Division, Saha Institute of Nuclear Physics, Kolkata, 700064 India

## Abstract

For the first time, the heat dried biomass of a newly isolated fungus *Arthrinium malaysianum* was studied for the toxic Cr(VI) adsorption, involving more than one mechanism like physisorption, chemisorption, oxidation-reduction and chelation. The process was best explained by the pseudo-second order kinetic model and Redlich-Peterson isotherm with maximum predicted biosorption capacity (*Q*_*m*_) of 100.69 mg g^−1^. Film-diffusion was the rate-controlling step and the adsorption was spontaneous, endothermic and entropy-driven. The mode of interactions between Cr(VI) ions and fungal biomass were investigated by several methods [Fourier Transform-Infrared Spectroscopy (FT-IR), X-ray Diffraction (XRD) and Energy-Dispersive X-ray spectroscopy (EDX)]. X-ray Photoelectron Spectroscopy (XPS) studies confirmed significant reduction of Cr(VI) into non-toxic Cr(III) species. Further, a modified methodology of Atomic Force Microscopy was successfully attempted to visualize the mycelial ultra-structure change after chromium adsorption. The influence of pH, biomass dose and contact time on Cr(VI) depletion were evaluated by Response Surface Model (RSM). FESEM-EDX analysis also exhibited arsenic (As) and lead (Pb) peaks on fungus surface upon treating with synthetic solutions of NaAsO_2_ and Pb(NO_3_)_2_ respectively. Additionally, the biomass could also remove chromium from industrial effluents, suggesting the fungal biomass as a promising adsorbent for toxic metals removal.

## Introduction

In recent years, the rapid increase in industrialization has emerged as a major problem due to the release of various heavy metals (like chromium, lead, arsenic) as waste directly into the surface of water-bodies^1^ making it unfit for human consumption^2, 3^. Given toxicity and related environmental hazards^4^, the concentration of these heavy metals in the effluent must be brought down to permissible limits before discharging into the surface water^5–7^. Unlike the existence of several heavy metals, the release of toxic chromium much beyond the permissible quantities was noticed in several countries and is one of the most impending issues in India^8^. Chromium pollution occurs predominantly from tannery industries which are located mostly in Uttar Pradesh and West Bengal, India^9, 10^. The effect of hexavalent chromium [Cr(VI)] is linked to neurotoxicity, genotoxicity, carcinogenicity and immunotoxicity^8, 11^. In the present scenario, the adverse effects of Cr(VI) to human health have been well documented^10^, that has drawn the attention of researchers in removing these from the contaminated sites. The conventional methods for metal removal are quite inefficient due to high operational costs and risk of secondary pollutants^12–16^.

Biosorption is a cutting-edge technology, which involves sorption of dissolved substances by a biosorbent which can significantly reduce the cost of chemical usage and pollution compared to the other traditional Cr(VI) removal processes^14, 17, 18^. Several investigations have been reported on metal binding efficiencies of various strains of bacteria, algae and fungi^4, 14, 19–26^. Among them, fungi are the best choice because of high cell wall material percentage and outstanding metal binding properties^2, 8, 14, 23, 27^.

We have isolated a new indigenous fungal strain *Arthrinium malaysianum* with high chromium removing efficacy. However, to date, the metal biosorption or biomineralization properties of the biomass of *Arthrinium sp*. have not been reported. Earlier, few *Arthrinium sp*. had been reported to be novel producers of many industrially important enzymes^28, 29^. *Arthrinium sp*. are non-pathogenic endophytes with immense pharmacological and medicinal applications^30^ that includes its antifungal and antioxidant properties^31^. Recently, we have illustrated the production of many important enzymes from this fungus *Arthrinium malaysianum* ^28^. In this respect, using fungal biomass for biosorption of toxic metals would add another dimension to the utility ﻿of this organism towards human welfare.

Here, we present the in-depth study that unravels the chromium adsorption behavior and mechanisms in the dried cell wall of *Arthrinium* *malaysianum*. It is a known phenomenon that living fungal cell walls are capable of reducing soluble Cr(VI) to insoluble Cr(III) to relieve its toxicity. However, by XPS analysis, the compounding effect was observed here exclusively on the heat dried fungal cell wall. Isotherm, thermodynamic and kinetic parameters were also estimated in describing the adsorption process. Many analytical methods (FT-IR, EDX, XRD) were employed to reveal the interactions between chromium ions and fungal cell wall while AFM and FESEM were adopted to investigate morphological changes of biomass exposed to Cr(VI). Box-Behnken statistical Design (BBD)^2, 8, 32^ has been employed to visualize mutual interactions among factors responsible for Cr(VI) adsorption process.

Single toxic metallic species rarely occur in natural wastewater, rather multiple metal ions occur in complex forms having interactive effects^13^. Thus, apart from chromium metal, we also checked the metal binding efficacy of our biomass for other two deadly heavy metals namely arsenic and lead. FESEM-EDX data confirmed the presence of both arsenic (As) and lead (Pb) peaks on the biomass surface warrants further mechanistic investigations in the near future. Therefore, the biomass of *Arthrinium malaysianum* could serve as a promising adsorbent for the removal of toxic heavy metals from aqueous solutions.

## Results and Discussion

### Identification of Cr(VI) adsorbing fungal strain

**Figure 1:**
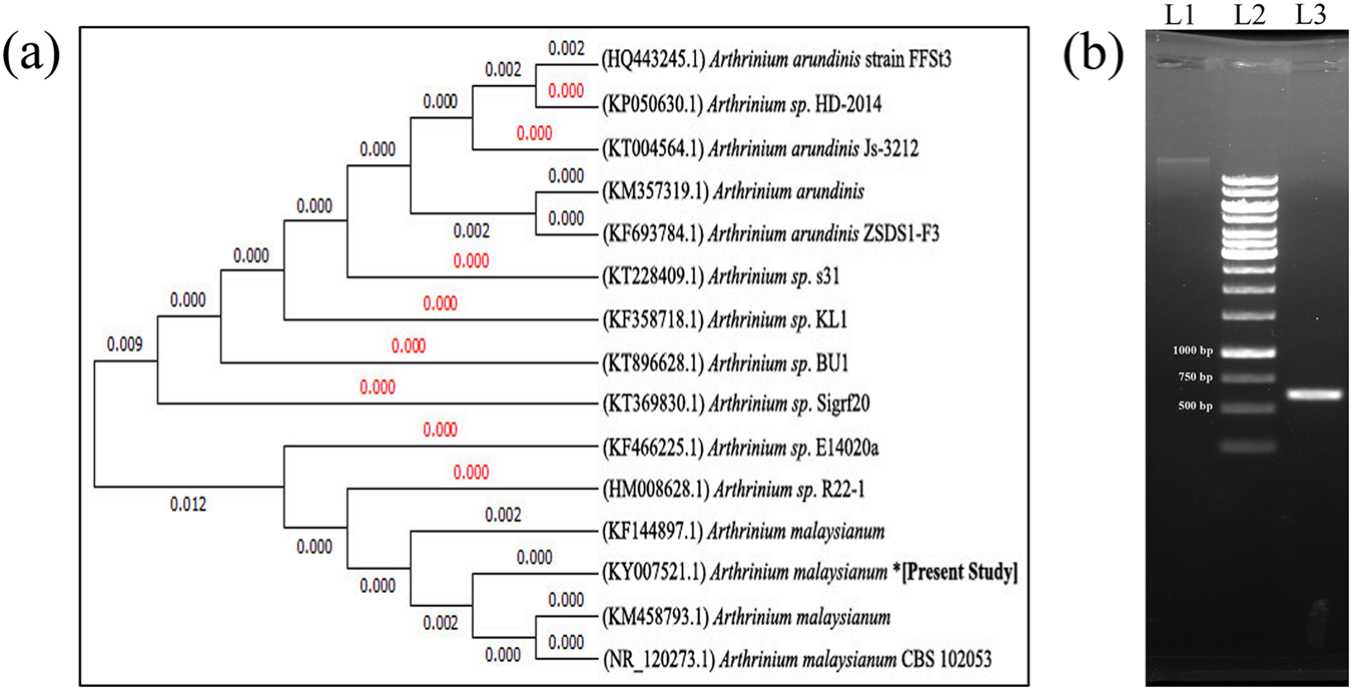
Molecular identification of the isolated fungus. **(a)** Neighbour-joining phylogenetic tree of *Arthrinium malaysianum* strain based on the nucleotide sequences of the ITS genes. The tree was constructed by Mega6 software. **(b)** *Arthrinium malaysianum* genomic DNA (L1), 1 kb DNA ladder (L2), and PCR product with amplicon size of 630 ± 8 bp (L3).

The 18S ITS gene sequence analysis of the fungal strain exhibited 99% homology with *Arthrinium malaysianum* (GenBank accession number: KF144897.1 and KM458793.1). The sequencing results were submitted to the NCBI GenBank database (Accession number: KY007521.1) and a Neighbour-joining phylogenetic tree (Fig. 1a) was constructed using Mega6 software (version 6.0). The ITS region of approximately 630 ± 8 bp of amplicon length was successfully amplified by PCR towards molecular fingerprinting study of this fungi (Fig. 1b) as it is considered to be the standard barcode for fungi identification^33–35^.

Endophytes like *Arthrinium sp*. represent a promising group of fungi because the genus *Arthrinium* is widespread in nature but less explored for many biological applications till date^28^. The idea of fabricating oven dried biomass (heat inactivated) of *Arthrinium malaysianum* (designated as AMB-*A* *rthrinium* *m* *alaysianum* biomass) was envisioned for noticeable bioremediation in a non-hazardous approach.

### Biosorption of Cr(VI) depends on pH and contact time: RSM study

In the present study, we have selected some important independent factors [A: contact time (h), B: biosorbent dosage (g L^−1^) and C: initial pH], after reviewing previous information^4, 6, 8, 17, 23, 27, 36^. It has been shown that the solution pH and contact time significantly influenced the responses (Table S1). Cr(VI) removal efficiency increased gradually with increase in biomass dose, and an optimal removal (~67%) was achieved with 4 g L^−1^ biomass in 20 h at pH 3.0 (Table S1). This may be due to increase in contact surface of adsorbent particles with increased biomass and more available binding sites for complexation of Cr(VI). At pH 3.0, hexavalent chromium exists in forms of Cr_2_O_7_^2−^ and HCr_2_O_4_^−^ ions and are adsorbed on positively charged sites through strong electrostatic force of attraction culminating in increased adsorption efficiency^2, 4, 8, 14, 37^. The equilibrium between the metal ions and the biomass was established at a biomass dose of 7 g L^−1^, only 8–10% increase in biosorption occurred with increase in biomass concentration to 8 g L^−1^. The 2^nd^ order polynomial model equation (shown below) was generated by Design-Expert software version 10.0 (see Methodology section for details):

$$\begin{array}{rcl}{\rm{Response}}\,({\rm{Y}}) & = & +\,60.37-3.08{\rm{A}}+0.94{\rm{B}}-22.39{\rm{C}}-5.00{\rm{AB}}-3.57{\rm{AC}}\\  &  & -\,0.32{\rm{BC}}-8.55{{\rm{A}}}^{2}-1.67{{\rm{B}}}^{2}-10.18{{\rm{C}}}^{2}\end{array}$$

where A, B and C correspond to independent variables of contact time (h), biomass weight (g) and pH, respectively. Percent of biosorption is the response (Y) here. We rejected the null-hypothesis by the model *F*-value (104.97) and probability (*p* < 0.05) from ANOVA test^2, 32, 38^. Based on the *p*-value (<0.05), we found two independent variables (A and C), one interaction term (AB), and two quadratic model terms (A^2^ and C^2^) were significant (Table S2a) in determining percent chromium biosorption^38, 39^. The calculated *R* ^2^ (0.9926) and adjusted-*R* ^2^ (0.9832) values were in reasonable agreement and we did not estimate the biased-*R* ^2^ value for our model. Adequate precision basically measures the signal (S) to noise (N) ratio, and S/N > 10 is desirable. In the present model, the S/N value was obtained to be 33.553 which indicated an adequate signal^38^. Other statistical parameters [standard deviation (2.31), mean (50.77), the coefficient of variance (4.56%)] had been summarized in Table S2b indicating the significance of our tested model^8, 38, 39^. Most of our experimental values were substantially correlated to the model predicted values with high regression coefficient (Fig. S1a), making it appropriate for a wide range of datasets^32, 38, 40^. As the biomass weight and contact time simultaneously increased, an increase in the Cr(VI) removal percentage was observed. Perturbation plot of the three independent factors has been shown in Fig. S1b. In the model, the center point, factors C (pH) shows a negative effect on response as it changes from the reference point. It was evident from the plot that, lower the pH, the higher the amount of Cr(VI) removal as shown by other researchers^2, 4, 8, 14, 38, 41^. Three response surface 3D plots were assessed in this study where the combined effects of contact time–biomass dose (Fig. S1c), pH–contact time (Fig. S1d), and pH–biomass dose (Fig. S1e) were demonstrated. We observed an elliptical 3D response surface plot in the combined effects of pH–contact time (Fig. S1d) indicated the interactions between these variables were very significant. The optimum levels of each variable in actual values (%, wv-1) were as follows: contact time = 20 h, biomass dose = 0.4 g (8 g L^−1^) and pH = 3, all of which were located within the experimental range. The experimental response under these optimum conditions was 70.8% which closely matched the yield predicted by the statistical model (72.16%) with *R* ^2^ value of 0.9926.

### Equilibrium isotherm studies showed dual mode of adsorption

**Figure 2:**
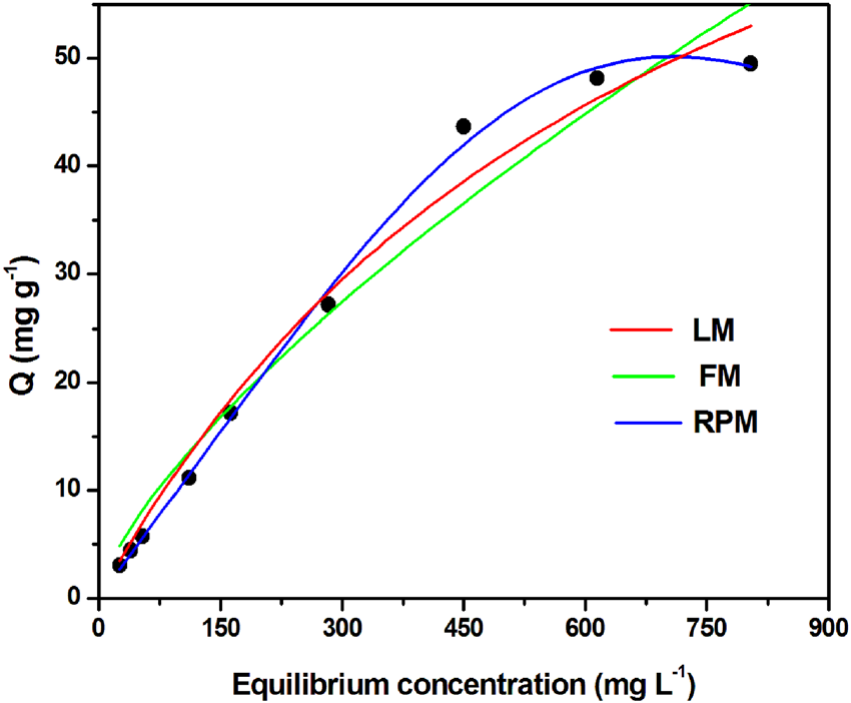
Equilibrium adsorption isotherm of chromium (VI) adsorption onto the biomass surface of *Arthrinium malaysianum*. Data representation as experimental outcome (●) fitted in Langmuir isotherm (), Freundlich isotherm (), and Redlich-Peterson hybrid () model. For each data acquisition, a known mass (~0.2 g) of heat inactivated fungal biomass was suspended in 25 mL of potassium dichromate solutions of concentration ranging from 50 to 1200 mg L^−1^ and kept under isothermal condition (30 °C) for 8 h.

Initially, we designed our study to evaluate the biosorption potential of the fungal biomass under varying concentrations (50 to 1200 mg L^−1^) of synthetic K_2_Cr_2_O_7_ solution. The Cr(VI) removal capacities were presented as a function of the initial concentration of Cr(VI). The experiments were done at constant pH 3.0, 8 g L^−1^ biomass dose and incubated at 30 °C temperature for 8 h. During biosorption, initial metal ion concentrations impart driving force which is indispensable for the mass transfer of metal ions from aqueous phase to solid phase. With increase in Cr(VI) ion concentration, the metal uptake (Q_*e*_) started increasing which reached to the maximum chromium loading capacity of ~48 mg g^−1^ of biomass when initial concentration was at 1000 mg L^−1^ (Fig. 2). Similar observations were reported by other groups^2, 18, 38^.

In equilibrium adsorption isotherm, values of the constants express the surface properties and affinity of the adsorbent sorption equilibrium, only when the concentration of sorbate in the bulk solution is in dynamic balance with that on the adsorbent surface. Based on the observation that the heat inactivated biomass has a clear ability in adsorbing chromium, we quickly ﻿worked on﻿ deciphering the type of adsorption. For checking the affinity of AMB for Cr(VI) ions, three most important isotherm models such as Langmuir, Freundlich and Redlich-Peterson dual isotherms were taken into account to analyze the actual data obtained from the process^42–44^. All calculated model parameters (obtained from Origin 8.0 program) are summarized in Table S3a. Usually, a lower χ^2^ (chi-square) and a higher *R* ^2^ value recommend a better fitting for isotherm curves^18, 45^. It could be seen from Fig. 2 that the equilibrium study gave reasonably good fits for all the models tested describing involvement of physisorption and chemisorptions processes. But best fitting was achieved with the Redlich-Peterson dual due to the highest regression coefficient (*R* ^2^ = 0.9987) and lowest Chi square (χ^2^ = 1.066) value (Table S3a). Generally, the value of Redlich-Peterson isotherm exponent (*β*) lies between zero (0) and one (1) but an adsorption model could be referred to as the Langmuir model when *β* is > = 1^45^. In present study, the *β* value of 3.166 thus confirms the adsorption mechanism to be a hybrid one, preferentially following the ideal monolayer adsorption coverage characteristics of Langmuir isotherm (*R* ^2^ = 0.9828 and χ^2^ = 7.34) with the predicted Q_*max*_ value of about 100.69 mg g^−1^of dry biomass. This data is substantially higher than other fungal biosorbents reported in literature for chromium^14, 46–60^.

### Adsorption kinetics was studied to understand the rate of reaction

**Figure 3:**
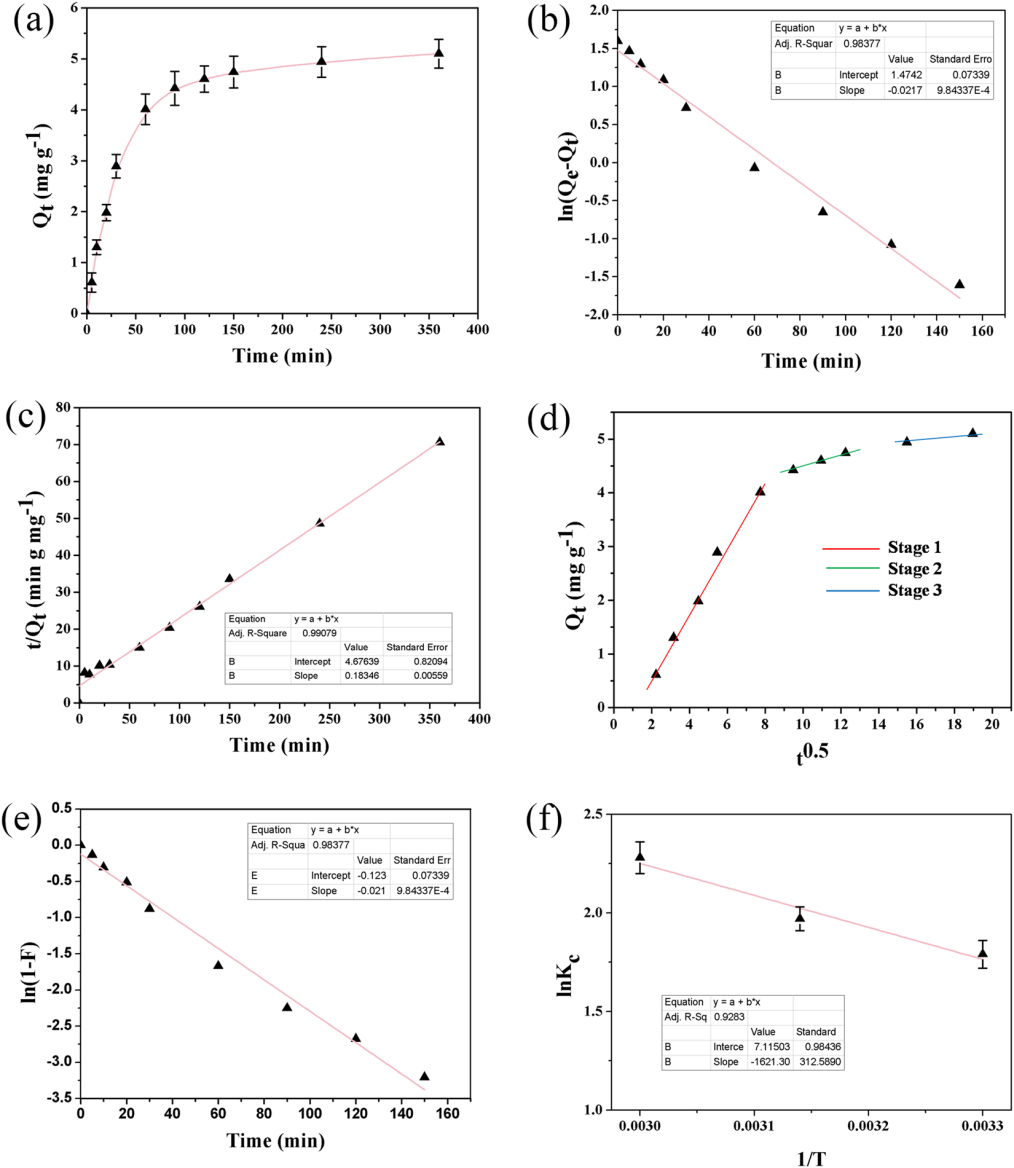
Adsorption kinetics and thermodynamics during chromium biosorption. (**a**) Effect of contact time on chromium (VI) uptake onto fungal biomass at experimental conditions (pH 3.0, Cr(VI) concentration of 100 mg L^−1^, biosorbent dose 8 g L^−1^), (**b**) pseudo-first order plot, (**c**) pseudo-second order plot, (**d**) intra-particle diffusion plot, (**e**) film diffusion plot, and (**f**) effect of temperature on adsorption. Data represent an average of four independent experiments; ±SD shown by the error bar.

We calculated the rate of the bioprocess through adsorption kinetics. Biosorption kinetics usually consists of multi steps which involve a rapid initial uptake of metal ions followed by mass transfer and relatively slower intra-particle diffusion. Figure 3 shows all the kinetic profiles for the adsorption of Cr(VI) by heat dried mycelia of *Arthrinium malaysianum*. To study this adsorption kinetics, fixed amount (8 g L^−1^) of biomass was incubated with potassium dichromate solution (100 mg L^−1^). The removal process consisted of two phases: a primary rapid phase of ~1.5 h which accounted for more than 75% of biosorption (~4.4 mg g^−1^) of the total metal (Fig. 3a). The kinetics of metal biosorption depends on the physical sorption on the cell surface and it is usually rapid during the early period of contact between sorbent and the sorbate. Subsequently, the second phase of biosorption reached equilibrium after approximately 6 h. Between 150 min to 350 min of contact, the increase in metal uptake was only 20–25% (4.4–5.1 mg g^−1^) respectively which could be explained through equilibrium phenomena^61^. To understand the detailed characteristics of the biosorption process, we also analyzed the behavior of the adsorption process by using different kinetic models like pseudo-first-order (PFO) (Fig. 3b), pseudo-second-order (PSO) (Fig. 3c), intra-particle diffusion (Weber-Morris) (Fig. 3d) and mode of film-diffusion (Fig. 3e). The above mentioned kinetic models were applied to fit our experimental data obtained from the batch experiments. The kinetic parameters and the determination coefficients (R^2^) were determined by linear regression and are given in supplementary information (Table S3b). It was shown that the PSO kinetic curves gave a better fit (R^2^ = 0.9907) to the experimental kinetic data than the PFO model (R^2^ = 0.9837), indicating the PSO kinetic model is much appropriate to describe the adsorption behavior of Cr(VI) onto biomass and occurrence of chemical adsorption^45, 62, 63^. The equilibrium capacity value (Q_*e*_ = 5.45 mg g^−1^) calculated using the PSO kinetic model (Fig. 3c) was found to be closest to the experimental value (5.10 mg g^−1^) indicating chemisorption as the rate limiting step for the removal of Cr^+6^ by the fungal biomass.

The intra-particle diffusion model (Fig. 3d) was plotted between the square root values of contact time and a solute adsorbed to check the effect of mass transfer resistance on the binding of Cr(VI) ions to the biosorbent. The plots also showed multi-linearity, thus indicating three steps of biosorption. Initial linear portion (red line) with little intercepts that nearly passed through the origin suggested that the intra-particle diffusion (IPD) may not be the rate-determining step but played an important role at this initial stage. The difusion of chromium from the solution to the dried cell surface of *A. malaysianum* resulted in a sharper slope (Fig. 3d). The second stage (green line) involved the slower adsorption phase implying rate-controlling phenomena and this becomes more evident for the intercepts of linear fit which increased or deviated from origin with an increase in contact time. We can have an idea about the boundary layer thickness through Weber-Morris plot assuming the fact that the intercept values are directly proportional to the boundary layer diffusion. Thus, the reason behind a deviation of the straight line from the origin in this plot might occur when there is variation in the rate of boundary layer diffusion at initial stage of adsorption. Finally in the third stage, (blue line) the IPD further slowed down due to the very low chromium concentration remained in the solution. Therefore, we may conclude that the whole adsorption process was mutually controlled by IPD and external mass transfer. Another crucial mechanistic aspect such as film diffusion with high regression coefficient could also be considered as the rate limiting stage in this bioprocess (Fig. 3e). Similar kind of observable facts was reported by other researchers^45, 64, 65^.

### Cr(VI) biosorption is endothermic and entropy driven

To explore the effectiveness of dried biomass in chromium bioaccumulation, thermodynamic parameters were determined. The thermodynamic parameters are shown in Fig. 3f and in Table S4. The negative values of ΔG° indicated thermodynamically feasible nature and spontaneity of the process. The negative ΔG° was found to increase due to any increase in temperature. This shows an increase in the feasibility of Cr(VI) biosorption at higher temperatures, which might be because of the higher temperatures cause the diffusion of Cr(VI) molecules from the solution to the biosorbents to be faster^36, 66^. This is also due to the increase in solubility of Cr(VI) ions. The positive ΔH° (23.47 KJ mol^−1^) indicates the endothermic nature of process whereas the positive value of ΔS° (60 J mol^−1^ kelvin^−1^) reveals the increased randomness at the solution-solid interface and occurrence of ion replacement reactions during the Cr(VI) biosorption onto the biomass. Basically, the heat produced during condensation process has similar order of magnitude as the heat of physisorption which lies between 2.1–20.0 KJ mol^−1^ while it is greater than 80 KJ mol^−1^ for the heats of chemisorption^67^. Hence, the chromium biosorption by fungal biomass would be attributed to a physisorption-chemisorption combination process rather than an ideal physical or chemical adsorption process.

### X-Ray diffraction studies revealed a change in structural integrity

**Figure 4:**
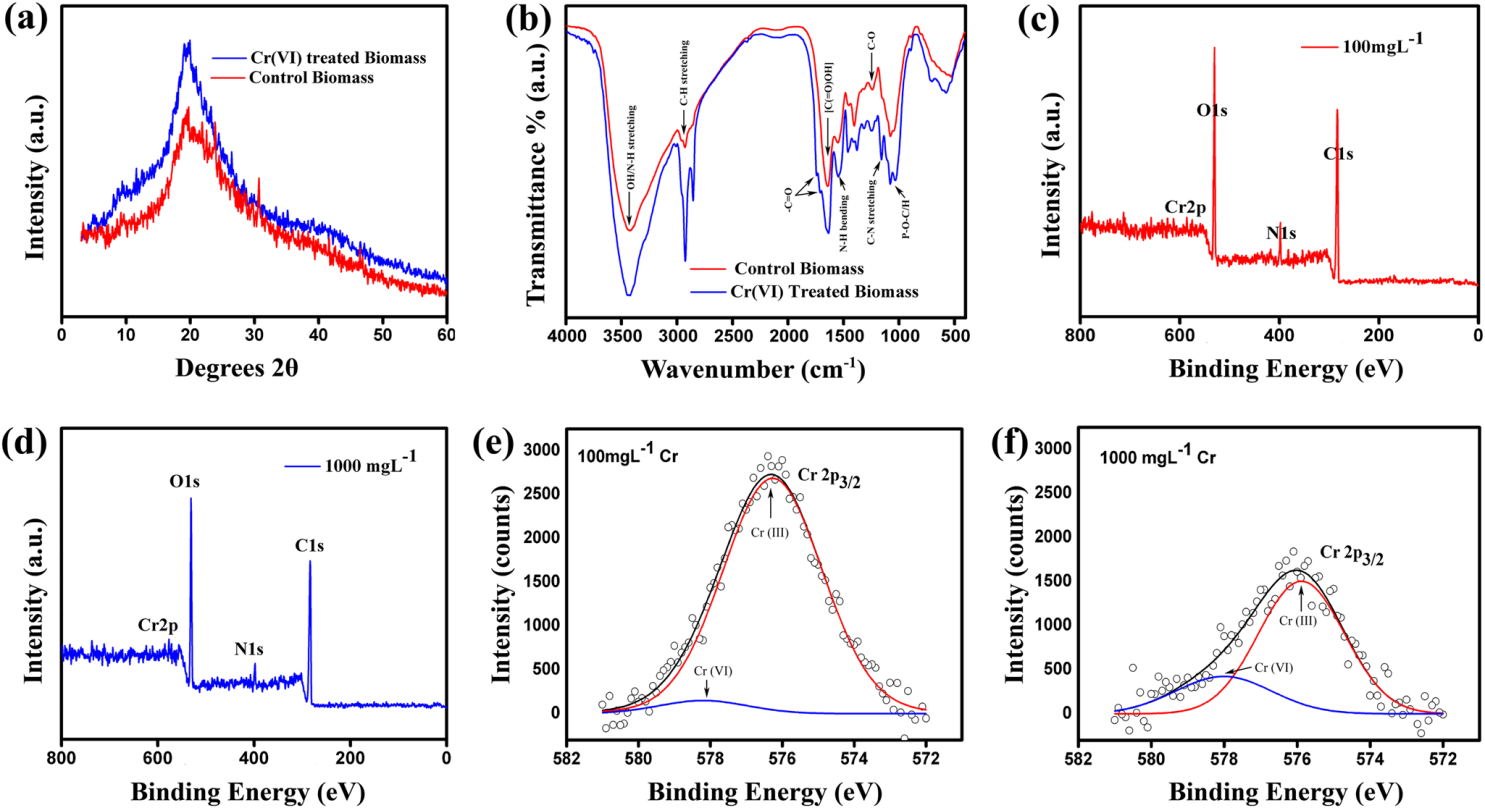
(**a**) Powder X-ray Diffraction (XRD) pattern of control () and chromium loaded () fungal biomass. (**b**) FTIR spectra of control () and chromium treated () biomass. (**c**) and (**d**) XPS wide scan spectra of 100 mg L^−1^ and 1000 mg L^−1^ of Cr^+6^ loaded biosorbent respectively. (**e**) and (**f**) Core level high resolution Cr2p_3/2_ spectra of 100 mg L^−1^ and 1000 mg L^−1^ chromium loaded *A*. *malaysianum* biomass respectively with representations of observed data (o), fitted data (**―**), Cr^+6^ () and Cr^+3^ ().

Control XRD data show that a small halo at 2theta angle of 10.5 characteristics of semi-crystalline nature of “chitosan” on the fungal cell wall^68^. In addition, a broad hump/peak conferred at about 19.5 which can be related to the amorphous nature (Fig. 4a). On treating with Cr(VI), relative intensity was substantially increased. Therefore, metal ions can be incorporated on the surface of dried AMB. Moreover, the crystallinity of the treated biomass increased compared to the semi-crystalline characteristic of the untreated biomass^69^. It may be concluded that the organized lattice of chitosan was distorted, due to their electrostatic interactions, and/or chelation.

### FT-IR Analysis revealed presence of functional groups on cell surface

The demonstrable results further insisted us to identify surface functional groups playing key roles in the bioprocess. FT-IR results confirmed that the main functional groups present on surface of heat pretreated *Arthrinium malaysianum* were amine, phosphate, hydroxyl and carbonyl (Fig. 4b). The FTIR spectra of pristine biomass (red line) showed many distinct peaks at 3422 cm^−1^ (representing range of 3500–3000 cm^−1^ due to –NH stretching in amides and O–H stretching vibration in alcohol and/or phenol), 2958 and 2927 cm^−1^ (C–H stretching vibrations of –CH_3_ and > CH_2_), 1642 cm^−1^ (C=O of protein bonds), 1549 cm^−1^ (N–H bending of amide II groups), 1453 cm^−1^ (C–H bending), 1404 cm^−1^ (C–N stretching), 1239 cm^−1^ (C–H stretching in amide III and C–O stretching) and at 1077 cm^−1^ for P–O–C and P–OH stretching (presence of phosphate groups)^70, 71^. The IR spectra of metal treated biomass (blue line) showed minute shriveling of –OH band and decreased intensity of C–O stretching band with a substantial increase in C=O band quantity and intensity evident from the appearance of new peaks at 1742 cm^−1^ and 1708 cm^−1^ compared to control biomass. This might be concluded that, during the interaction of Cr^+6^ to fungal biomass, few C–O groups were oxidized into C=O entities^71^ resulting in a reduction of Cr^+6^ to Cr^+3^. Very similar observations were reported by other researchers^72–74^. The FT-IR spectra of the biomass exposed to Cr(VI) ions also exhibited shifts of the characteristic bands 1642 cm^−1^ to 1635 cm^−1^ and 1549 cm^−1^ to 1547 cm^−1^. In control sample, we got a distinct peak at 1404 cm^−1^ which disappeared in metal loaded sample, attributed to the complexation of phosphate and sulfonyl group’s coordination with chromate ions. Results implied not only involvement of amine and carbonyl groups in biosorption of metal ions, but also the possible occurrence of ion-exchange and/or complexation process^25^. The wave number ranging from 1000–1400 cm^−1^ represented sulfur and phosphorus compounds, more specifying sulfonamides and N-substituted sulfonamides of solid samples, phosphate esters (at 1200–1400 cm^−1^). Thus the peaks at 1379 cm^−1^ and 1246 cm^−1^ in Cr(VI) laden samples represented sulfonyl and phosphate ester bonds of polysaccharide respectively. Moreover, a shift in peak from 1029 cm^−1^ to 1033 cm^−1^ might represent the formation of Cr(III)-phosphate compound^75^. The characteristic band region at 450–650 cm^−1^ suggested the presence of nitro (–NO_2_) compounds and sulfide groups. The frequency ranging from 400–550 cm^−1^ and 550–700 cm^−1^ also denotes disulfide and sulfide groups respectively^14^. The shift in the peak from 531 cm^−1^ for pristine biomass to 576 cm^−1^ upon metal exposer, specifies contribution of nitro compounds and disulfide groups in *Arthrinium malaysianum* biomass surface. This region might be attributed to the formation of Cr(OH)_3_ as described by previous researcher^37, 71^. FTIR peaks are assigned and shown in tabular form (Table S5). We thus propose that, during this bioprocess, the anionic Cr(VI) readily interacted to the hydroxylated functionalities (C_x_OH) which served as a reducing substrate^72^. This reduction phenomenon of Cr(VI) to Cr(III) was further confirmed by XPS.

### XPS studies confirmed the presence of two chromium species [Cr(VI), Cr(III)] and reduction of Cr(VI) to Cr(III)

X-ray photoelectron spectroscopy (XPS) is a very powerful method for surface characterization. It gives information about the chemical state, chemical linkage, chemical composition as well as atomic ratio of a sample. Figure 4c,d represent the survey scans of chromium laden (100 and 1000 mg L^−1^ of K_2_Cr_2_O_7_ solutions respectively) fungal biomass. The survey scan is a rapid wide scan which gives instant information about the type of elements present in the sample. Our survey scan data showed the presence of Cr, N, O and C elements evident from their characteristic binding energy (eV) peaks^13, 37^. The presence of chromium peak further directed us to decipher the chemical properties of chromium adsorbed on biomass surface. Two symmetric peaks due to the Cr2p core level appeared in the chromium-loaded mycelia (Fig. 4e and f) at 576.3 and 578.4 eV, corresponding to Cr(III) and Cr(VI) species, respectively. The symmetric peaks corresponding to the Cr2p core level at 576.3 eV indicated the formation of chromic hydroxide^37^. Thus it might be concluded from both FT-IR and XPS data that Cr(VI) initially binds to the protonated groups and then reduces to Cr(III), and consequently binds to the available carboxyl groups of the mycelia. However, it is worth mentioning here that, with an increase in chromium concentration [high chromium loaded mycelia (1000 mg L^−1^)], the intensity of the Cr2p peak at higher energy (578.4 eV) increased compared to that present at lower energy (576.3 eV). This indicated that at much higher chromium concentration, reduction of Cr(VI) to Cr(III) by biosorbent was less. It was noted that sorption of Cr(VI) with Cr(OH)_3_ matrix actually favored our experimental condition, where the electrostatic attractions took place between positively charged Cr(OH)_3_ and negatively charged chromate ions (Fig. 4e) further supported the FT-IR data. These results are similar to the Das *et al.* work^37^ where the mechanism of chromium interactions with *Aspergillus versicolor* fungus has been carried out. The spectral position of Cr2p level at 576.3 eV showed a tiny shift in high chromium-loaded (1000 mg L^−1^) biomass, culminating to a characteristic peak of Cr_2_O_3_ (~576.0 eV) to Cr(III) (Fig. 4f). Similar observations were reported by Vieira *et al*.^41^ affirming the predominant signature of Cr(III) species.

During XPS studies, UHV graded double sided carbon tapes were used for samples mounting purposes. Since, theses tapes contain carbon (C) and oxygen (O) along with O atoms in chromate ions, we did not record spectral profiles corresponding to C1s and O1s. In fact, this study has been done with an aim to comprehend the fate of toxic hexavalent chromium on binding to the fungal surface and its ability to convert the Cr^+6^ to non-toxic Cr^+3^.

### Changes in cellular microstructure were evident from FESEM-EDX studies

**Figure 5:**
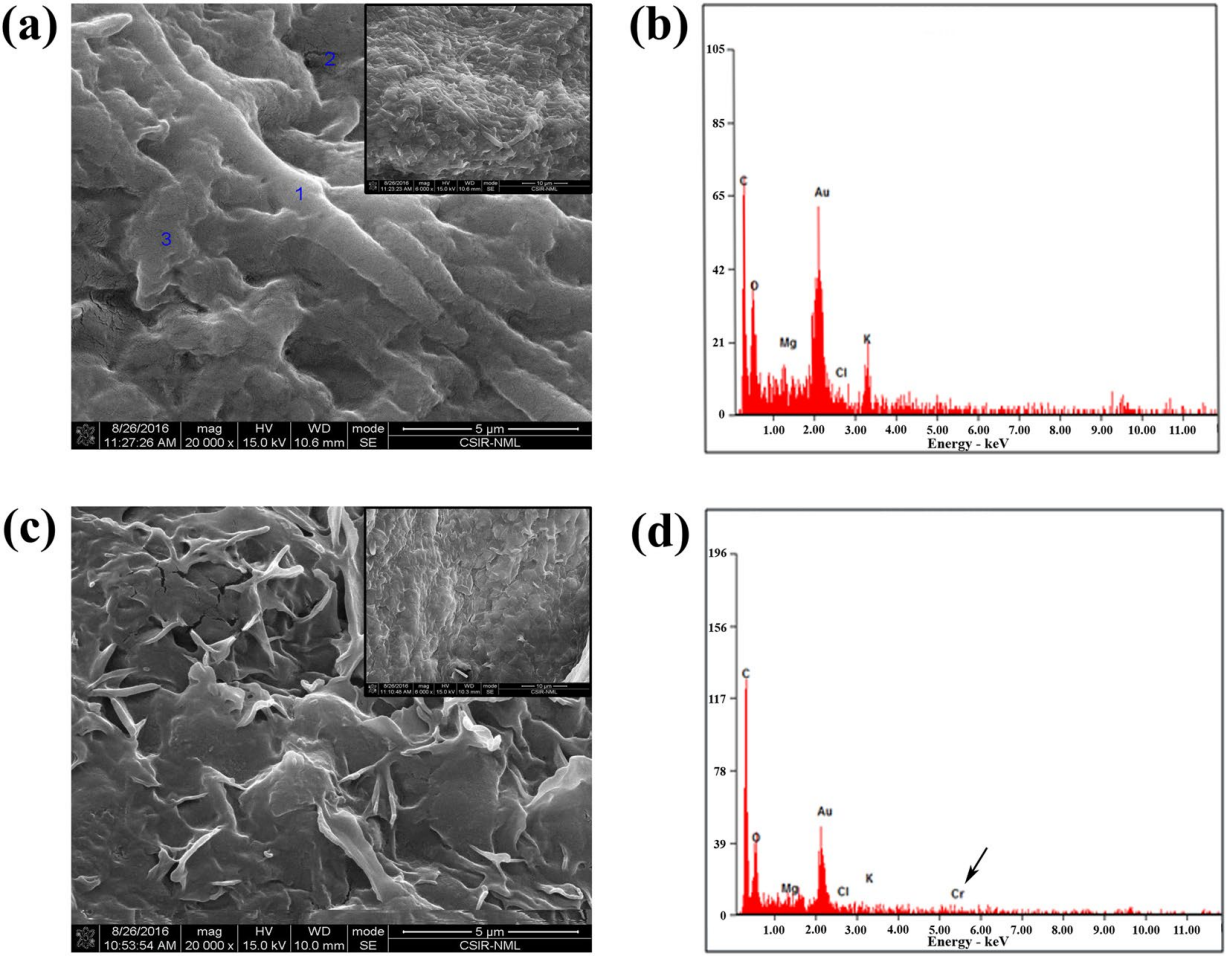
FE-SEM images of the surface of heat dried *A*. *malaysianum* biomass and EDAX spectra. (**a**) SEM micrograph before chromium adsorption by biomass, (**b**) corresponding EDAX spectra without any chromium signature, (**c**) SEM micrograph showing the deposition of tiny, spherical particles onto the surface of fungal biomass after binding to chromium, (**d**) typical EDAX spectra of *A*. *malaysianum* biomass after Cr(VI) loaded indicated by arrow.

SEM micrographs (20,000× magnification) were obtained before (Fig. 5a) and after Cr(VI) biosorption (Fig. 5c) onto heat treated biomass. The FESEM-EDX analysis made the Cr(VI) adsorption more visual between biosorbent and metal ions. Deposition of tiny, spherical particles making the surface rough was apparent on the biomass surface. The total scanned area seemed to be saturated with chromium. This observation was further confirmed by the EDX analysis which revealed Cr peaks in the spectra (Fig. 5d) while no such peak was observed on the surface of biomass confirming the deposition of chromium (Fig. 5b). Similar type of observation was reported by Ramrakhiani *et al*.^14^ and Das *et al*.^71^.

### AFM imaging demonstrated the change in surface structure integrity

**Figure 6:**
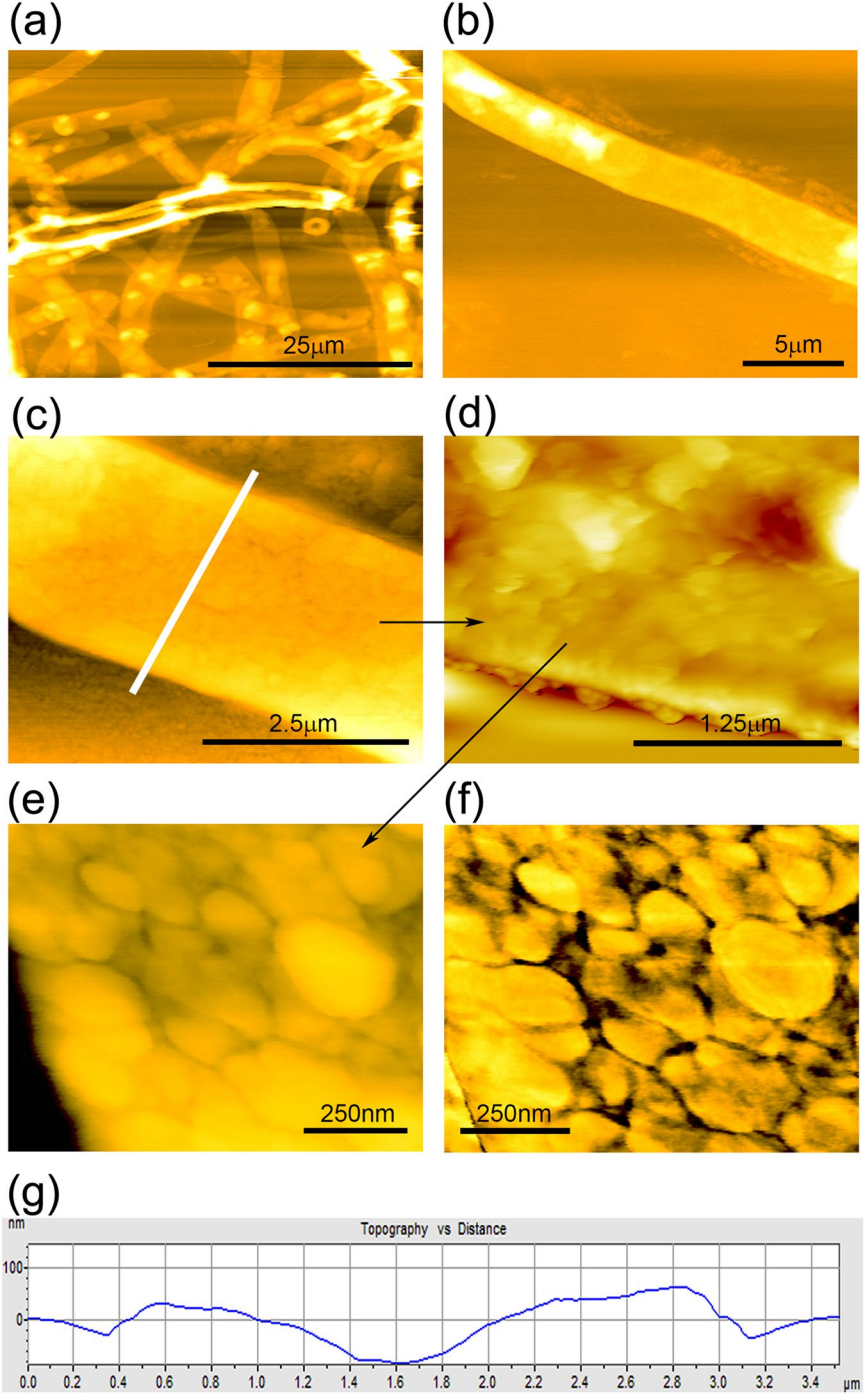
AFM images of the surface of heat dried *A*. *malaysianum* biomass. (**a**–**e**) Topographic AFM images of control biomass (before chromium adsorption) showing relatively smooth surfaces of the fungal mycelia with some consistent protuberant structures. (**f**) Phase AFM image of (**e**) showing rounded and textured pattern with pore-like distribution. (**g**) Cross-sectional analysis of (**c**) image from the portion marked with white line.

**Figure 7:**
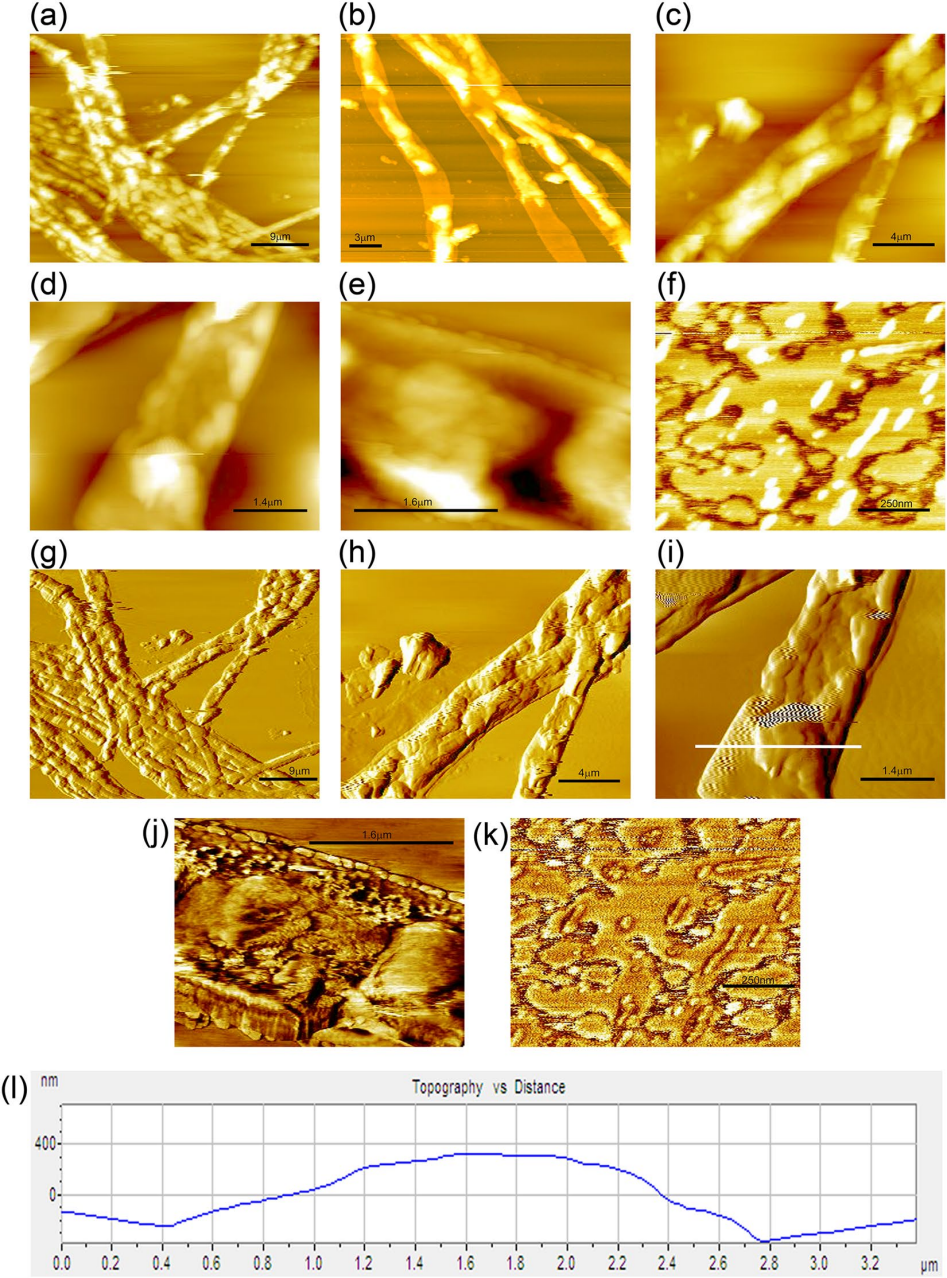
Topographic (**a**–**f**) and phase (**g**–**k**) contrast AFM images of chromium adsorbed *A*. *malaysianum* biomass indicating a change of the surface morphology into a regular cage-like pattern due to the adsorption of chromium on cell surface. (**l**) Sectional analysis of (**i**) image from the segment marked with white line.

AFM is another powerful imaging technique by which surface ultra-structure and properties can be resolved successfully. Hence in the present work, we have tried to incorporate AFM imaging (both topographic and phase mode) to visualize the morphological differences between control biomass (Fig. 6) and metal loaded biomass (Fig. 7). Initially, we captured images by scanning a 25 *μm* × 25 *μm* area where fungal mycelia were found as clusters (Fig. 6a). We gradually reduced the image size to isolate a single mycelium with higher resolution (250 nm × 250 nm) and before imaging, scanning was done in both forward and backward directions to negate any possible alteration in imaging (hysteresis). Initially, low resolution topographic images (Fig. 6a–c) revealed relatively smooth surfaces of the fungal mycelia without any noticeable structural characteristics, but higher resolution images showed some consistent protuberant structures emerged on the fungal cell wall and the surface was found to be more textured (Fig. 6d,e). Rounded and textured pattern with the pore-like distribution of mycelia was evident from the topographic (Fig. 6e) and its corresponding phase image (Fig. 6f). Sectional analysis of this mycelial surface (Fig. 6c, indicated by white line) clearly demonstrated the homogeneously distributed textured pattern throughout the cell wall (Fig. 6g) having approximately 75 nm of average height.

In contrast, structural alteration after metal deposition has been depicted in Fig. 7. It is evident that a larger amount of chromium is adsorbed onto the cell surface that resulted in the bulging. Chromium adsorption could have altered the biosorbent configuration by creating more favorable condition for capturing metal ions. Metal deposition on the fungal cell mass was observed both in topographic (Fig. 7a–f) as well as phase images (Fig. 7g–k). There was a transformation of the surface morphology into a regular cage-like pattern (Fig. 7f) with densely arranged layer of the metal ions (confirmed by EDX) on cell surface. In metal treated mycelia, we could not observe the segregated domains with rounded features that we noticed on the surface of untreated control mycelia. Figure 7e depicted the appearance of few dents on cell surface which created undulated structures on it. Sectional analysis (Fig. 7l) demonstrates the substantial increase in height profile (~390 nm) in metal loaded biomass compared to untreated control one (~75 nm).

AFM phase imaging is an indispensable method by which sample heterogeneity can be measured and more information can be obtained from phase images than corresponding topographic images. An obvious increase in surface roughness of the chromium loaded mycelia (Fig. 7) in comparison with that of the control mycelia (Fig. 6) may result in poor image resolution due to tip-sample interaction, resulting in inconsistent response^37^.

### FESEM-EDX of Arsenic and Lead adsorption on fungal biomass

**Figure 8:**
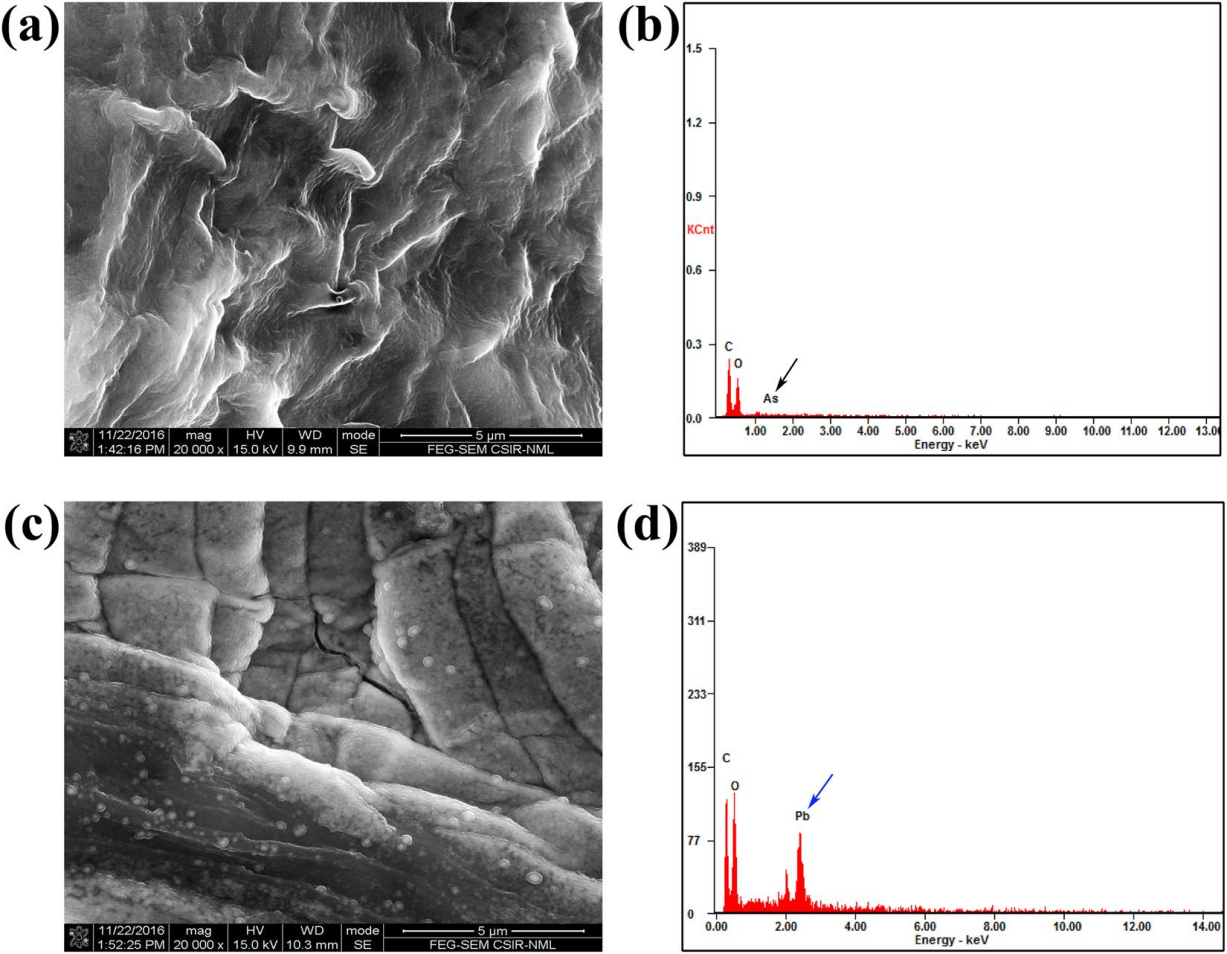
FE-SEM and EDX analysis of the surface of *A*. *malaysianum* biomass after arsenic and lead adsorption. (**a**) FE-SEM micrograph after arsenic (As) adsorption by biomass, (**b**) corresponding EDAX spectra with arsenic signature indicated by black arrow, (**c**) FE-SEM image showing the formation of shiny coating the surface of the fungal biomass after binding of lead, (**d**) subsequent EDAX spectra confirming the presence of lead (Pb) peak shown by blue arrow.

To check the efficiency of this organism on Arsenic (As) and Lead (Pb) removal, a pilot study was done in which 1% (w/v) solutions of sodium arsenite (As^+3^) and lead nitrate (Pb^+2^) were taken and incubated with dried biomass (6 g L^−1^) for 10 h. Here, we followed the same procedure of FESEM-EDX sample preparation as we did in case of chromium. Characteristic changes in surface structures were observed under SEM imaging for arsenic treated (Fig. 8a) and lead treated (Fig. 8c) biomass compared to the control (Fig. 5a). Some spherical particles adsorbed on the surface of the organism were more prominent in case lead treated sample (Fig. 8c). Metal adsorption was further supported by the EDX spectra of the As (Fig. 8b) and Pb (Fig. 8d) which showed an increased accumulation of Pb particles suggesting, enhanced efficiency of the organism to remove lead^76^. This study is underway and gives scope to other researchers too, to explore this organism in removing As and Pb, that are important waste water pollutant/contaminants of the system.

### Desorption studies showed that most of the bound chromium on *A*. *malaysianum* surface was in trivalent form

NaOH was most efficient eluant that could desorb ~41.17% of adsorbed Cr^+6^ (2.705 mg g^−1^ desorbed metal) from fungal biomass after 24 h. We could recover high amount of Cr^+6^ as the predominant species of chromium on biomass surface that was in the form of Cr^+3^ which could be oxidized by a strong base. These outcomes also corroborated to the XPS results where we showed an abundance of Cr^+3^ on biomass surface.

### *A. malaysianum* biomass can also remove chromium from leather industry effluent

It was found that the biomass could efficiently remove (~30% removal at effluent pH and ~70% removal at pH 3) hexavalent chromium from aforesaid effluent. Results also suggested that this removal efficiency was closely similar to monometallic system (chromium alone) and not hindered by the presence of other metals (like Fe, Cu, Ni, Zn etc.) found in effluents^76^.

## Conclusion

In summary, the renewable heat dried biomass of *A*. *malaysianum* fungus was used as efficient biosorbent for the removal of toxic Cr^+6^ from synthetic solution as well as from industrial effluents. The maximum experimental adsorption capacity (Q_*max*_) of the biomass was found to be 100.69 mg g^−1^, followed a hybrid adsorption model of Cr^+6^ with a preference for Langmuir isotherm. The Q_*max*_ value was higher than other fungal biosorbents reported for chromium (Results and Discussion section). The Cr^+6^ depletion was greatly increased with decrease in pH and increase in contact time, temperature and biomass weight. This endothermic, spontaneous bioprocess involved more than one mechanism like physisorption, chemisorption, oxidation-reduction as well as chelation. Film diffusion was considered to be the rate-limiting step while chemisorption phenomenon was described by the pseudo-second order kinetic model. Through XPS analysis, we reported that the heat dried biomass could reduce Cr^+6^ to Cr^+3^ irrespective of chromium concentrations. Here we successfully employed FESEM, AFM and XRD to resolve the surface structure changes during adsorption. FT-IR data confirmed the binding of chromate to protonated active sites with subsequent reduction to Cr^+3^ species by hydroxyl and carbonyl groups and oxidation of C–O to C=O. In addition, the biomass mediated chromium removal from industrial effluents also throws light on the possible commercial application. In future studies, we intend to utilize the fungal biomass efficiently for the synthesis of metallic nano particles during biosorption. From a practical viewpoint, these results could undoubtedly lead to a deeper understanding of the direct contribution of the newly isolated fungus in bioremediation.

## Materials and Methods

### Materials

Analytical grade potassium dichromate (K_2_Cr_2_O_7_), Sodium arsenite (NaAsO_2_), and lead nitrate [Pb(NO_3_)_2_] were purchased from Sigma-Aldrich, India. A stock solution (1000 mg L^−1^) of hexavalent chromium was prepared by dissolving exact quantities of K_2_Cr_2_O_7_ in deionized water (Milli-Q Millipore 18.2 MΩ cm^−1^). Cr(VI) was measured by standard 1,5-diphenyl carbazide (DPC) method^14, 72, 77^. Total chromium concentration was estimated through atomic adsorption spectrometry^36^. Lead and arsenite solutions (1%, w v^−1^) were also prepared in Milli-Q water. GeneJET genomic DNA purification kit (#K0721) was purchased from Thermo scientific. All PCR primers were customized from IDT, Inc. QIAquick PCR purification kit (Cat. No. 28104) was used for PCR clean-up purposes. All the other chemicals were of analytical grade.

### Isolation and molecular identification of the fungus

A novel fungus was isolated as a contaminant from the growing mat of mushroom *Termitomyces clypeatus*. The fungus culture was maintained on the potato dextrose agar (PDA) slants and stored at 4 °C. The fungus was routinely cultured in a filamentous form under submerged condition (150 rpm) for 3 days at 28 °C in yeast extract peptone glucose (YPG) media, which consists of yeast extract of 3 g L^−1^, peptone 5 g L^−1^ and dextrose (α-D-glucose) 20 g L^−1^. Media pH was adjusted to 5.0.

Details of the ITS-based molecular identification of the fungus have been given elsewhere (see supplementary information). PCR conditions have also been depicted in tabulated form in Table S6a,b.

### Preparation of functionalized biomass

For preparing the dead biomass of the fungus, an appropriate amount of live biomass was autoclaved. The heat inactivated *Arthrinium malaysianum* biomass (AMB) of was obtained by filtering the culture medium and the mycelia were washed thoroughly with deionized water. It was kept at 50 °C for 20–22 h or until it became crispy. The dried biomass was stored in an airtight container for further use.

### Cr(VI) biosorption efficacy of heat dried fungal biosorbent

These experiments were done by using 25 mL of Cr(VI) test solution (100 mg L^−1^) in Erlenmeyer flasks incubated at 30 °C at pH 4.0. The desired biomass dose was added to the flasks and kept under shaking (150 rpm) condition. After completion, solutions were separated from biomass through vacuum filtrations with Millipore membrane. Total metal concentrations were estimated by atomic adsorption spectroscopy (AAS). Efficiency (R) of metal removal [percentage of hexavalent chromium adsorbed] by the biomass as equation (1):

1 $$Efficiency\,(R)=\tfrac{{C}_{i}-{C}_{e}}{{C}_{i}}\times 100$$

where C_e_ and C_i_ are the equilibrium and initial metal concentrations respectively. The amount of adsorbed Cr(VI) per gram biomass was obtained using equation (2):

2 $${Q}_{e}=V({C}_{i}-{C}_{e})/M$$

where, Q_e_ = metal uptake [mg Cr(VI) g^−1^ of biomass]. C_i_ = initial metal ion concentration (mg L^−1^); C_e_ = metal ion concentration after biosorption (mg L^−1^); M = weight of biomass (g); V = Volume of metal solution (mL).

### Equilibrium adsorption isotherm

To evaluate the equilibrium data for biosorption of Cr(VI) onto dried AMB, the most well studied isotherm models such as Langmuir, Freundlich and Redlich-Peterson were employed^42–44^ (See supplementary information for the experimental details). The equations of the above isotherms are shown in Table S7.

### Adsorption Kinetic study

The adsorption kinetic data were analyzed using various models like pseudo-first order, pseudo-second order, intra-particle diffusion (Weber-Morris) and film diffusion^25, 65^. Details of the experimentations have been given in supplementary Information.

### Batch biosorption experiments using Response Surface Methodology (RSM)

Batch experiments were designed statistically through RSM^2, 8, 32^ to study the effects of important parameters that had an influence on biosorption. The effect of initial pH (3–5), biomass dose (4–8 g L^−1^) and contact time (16–24 h) onto the Cr(VI) removal were analyzed using statistical models. The Box-Behnken design for these three independent variables, each at three levels, was used to develop a second-order polynomial model for determining further optimum levels, presented in Table S8. A total of 17 experimental runs with various combinations of contact time (A), biomass dosage (B) and initial pH (C) were conducted. The behavior of each variable, their interactions, and statistical analysis to obtain predicted responses was explained by the following second-order polynomial equation (3):

3 $$Y={\beta }_{o}+\sum {\beta }_{i}{x}_{i}+\sum {\beta }_{ij}{x}_{i}{x}_{j}+\sum {\beta }_{ii}{{x}_{i}}^{2}$$

where Y is the predicted response, *β*_o_ - offset term, *β*_i_ - linear effect, *β*_ii_ - squared effect, *β*_ij_ - interaction effect, and x_i_ is the i^th^ independent variable. x_i_x_j_ and x_i_^2^ represented the interaction and quadratic terms, respectively followed by the quadratic model verification^2, 8^. For details, see supplementary Information.

### Determination of Thermodynamic parameters during chromium adsorption

The thermodynamic behavior of chromium biosorption was studied at three different temperatures (30 °C, 45 °C and 60 °C) as described previously^42^. The thermodynamic parameters like change in Gibb’s free energy (**∆** *G°*), enthalpy (**∆** *H°*), and entropy (∆*S°*) were also calculated. Detailed experimentations are described in the Supplementary Information section.

### Chromium interaction with *A*. *malaysianum* oven dried biomass: investigation with various microscopic and biophysical studies

#### FESEM-EDX analysis

Investigation of surface morphology and microstructure of the fungal biomasses (untreated and metals laden) were monitored using field emission scanning electron microscopy (FEI-Nova Nano SEM 430 operated at 15 kV) and elemental compositions were established by energy dispersive X-ray analysis (EDX). Experiments were carried out by incubating 0.2 gm of dried biomass in 25 mL K_2_Cr_2_O_7_ solution for 10 h^78, 79^. The electron beam acceleration tension was fixed to 15.0 KV, the take-off angle was 35.0°, and the acquisition time for the spectrum was 20 s. In order to compare element concentrations among different positions, each position area for EDX analysis was set to approximately 0.25 µm × 0.25 µm^6^.

#### Immobilization of fungal mycelia for atomic force microscopic (AFM) imaging

The dried fungal mycelia were grounded to obtain uniform sized particles followed by sieving through 150 mess sieve. In brief, 0.2 g of this powdered biomass was mixed with 1 mL of 10 mM phosphate buffer (0.22 filtered) and incubated for 8–10 h at 4 °C. Next, the mixture was macerated in a previously chilled mortar-pestle (‘agate’ material) to make a uniform pasted liquid. Later, this paste was further diluted (approximately 500–600) in autoclaved MQ water (0.22 filtered) and then incubated with freshly cleaved mica sheet (ASTM V1 Grade Ruby Mica from MICAFAB, Chennai) for 30 min, followed by frequent washing with deionized water (Milli-Q Millipore 18.2 MΩ cm^−1^) to remove loosely attached mycelia. The mica sheet was then mounted for AFM study after air-drying. The surface topography and nano-mechanical properties of the metal treated mycelia were compared with those of the pristine mycelia^23, 37^. Images were recorded in air at ambient condition in AFM (Veeco Metrology, Autoprobe diCP-II, Model No AP0100), and in tapping mode for minimizing sample damage by the scanning tip (cantilever: 0.01–0.025 Ω cm antimony doped silicon). The cantilever with long tips (aspect ratio 4:1) and spring constants ranging from 40 N/m and resonance frequencies of 290 kHz, was used to image the surface morphology of the mycelia. The force applied by the scanning tip was controlled to avoid dissection of the samples which were scanned in both front and back directions several times before recording an image. Typically, we began by scanning 50 μm × 50 μm areas and then gradually reduced the scan area to isolate individual cells^23, 37^.

#### Powder X-ray Diffraction (XRD)

Crystallization of the pristine and metal laden AMB was determined by X-ray powder diffractometer (RIGAKU, model ULTIMA-III, Japan) equipped with Cu-Kα (λ = 1.54056 Å) radiation with scan speed of 5° min^−1^ and in a 2θ range of 5–60^42, 65^.

#### Fourier Transform Infrared Spectroscopy (FT-IR)

FTIR spectra (region 4000–400 cm^−1^) of heat dried as well as chromium laden biomass (as described in section 2.8) were obtained by using JASCO FTIR instrument-410. The samples were pressed into spectroscopic quality KBr pellet with a sample/KBr ratio about 1/100.

#### X-ray photoelectron spectra analysis

XPS core-level spectra of the *A*. *malaysianum* mycelia before and after sorption were recorded by an Omicron Multiprobe (Omicron NanoTechnology GmbH, UK) spectrometer fitted with an EA125 hemispherical analyzer. A low-energy electron gun (SL1000, Omicron) with a large spot size was used to neutralize the sample. The potential of the electron gun was kept fixed at −3 eV on the ground potential. A monochromated Al-Kα x-ray source operating at 150 W was used for the experiments. The pass energy of the analyzer was fixed at 20 eV for all the scans. The binding energies (BE) were calibrated against the C1s peak at 285.0 eV. The curve fitting was done by a program on mixed Lorentzian-Gaussian function^37, 80, 81^ with a linear background to determine the binding energy of the element core levels.

#### Reusability of the biomass

Desorption experiments were carried out following earlier methods^8, 82^ with little modification. Experimental details have been given in supplementary information section.

#### Treatment of effluent from leather industry

The chromium removal efficiency of the biomass was also checked by procuring CETP-inlet effluent (Common Effluent Treatment Plant-inlet area) from leather industry as a feed solution. The experiment was carried out first at the recorded pH level of the effluent (pH 7.3 ± 0.9) and also at a pH adjusted to 3.0 (Table S9). Fungal biomass (8 g L^−1^) was incubated with waste water at ambient temperature for 24 h under shaking condition.

#### Statistical analysis

All experimental results were expressed as mean data from triplicate sets, considering the *p* value is less than 0.05 using Design-Expert 10.0. The magnitude of regression coefficient and Chi-square were obtained using Origin 8.0 program.

## Electronic supplementary material

Depletion of Cr(VI) from aqueous solution by heat dried biomass of a newly isolated fungus Arthrinium malaysianum: A mechanistic approach
