# Supplementary material for: Depletion of Cr(VI) from aqueous solution by heat dried biomass of a newly isolated fungus Arthrinium malaysianum: A mechanistic approach
Source: Sci Rep. 2017 Sep 12;7:11254. doi: 10.1038/s41598-017-10160-0 (PMC5595784; doi:10.1038/s41598-017-10160-0)
Supplement: Supplementary file 1 — Depletion of Cr(VI) from aqueous solution by heat dried biomass of a newly isolated fungus Arthrinium malaysianum: A mechanistic approach [file 41598_2017_10160_MOESM1_ESM.pdf]

Electronic Supplementary Information

Depletion of Cr(VI) from aqueous solution by heat dried biomass of a newly isolated fungus *Arthrinium malaysianum*: A mechanistic approach

Rajib Majumder[1], Lubna Sheikh[1], Animesh Naskar[2], Vineeta Verma[1], Manabendra Mukherjee[3] and Sucheta Tripathy[1*]

[1]Structural Biology & Bio-Informatics Division, CSIR-Indian Institute of Chemical Biology, Kolkata-700032, India, [2]Department of Food Technology and Biochemical Engineering, Jadavpur University, Kolkata-700032, India. [3]Surface Physics and Material Science Division, Saha Institute of Nuclear Physics, Kolkata-700064, India,

Supporting Information contains detailed description of some experimental procedures with some additional figure and tables. This consists of 14 pages including this one. There is 1 Figure (Fig. S1), 9 Tables (Table S1-S9) and 1 Graphical Abstract.

*Corresponding author: **Dr. Sucheta Tripathy**
E-mail: tsucheta@iicb.res.in
tsucheta@gmail.com

Graphical Abstract:

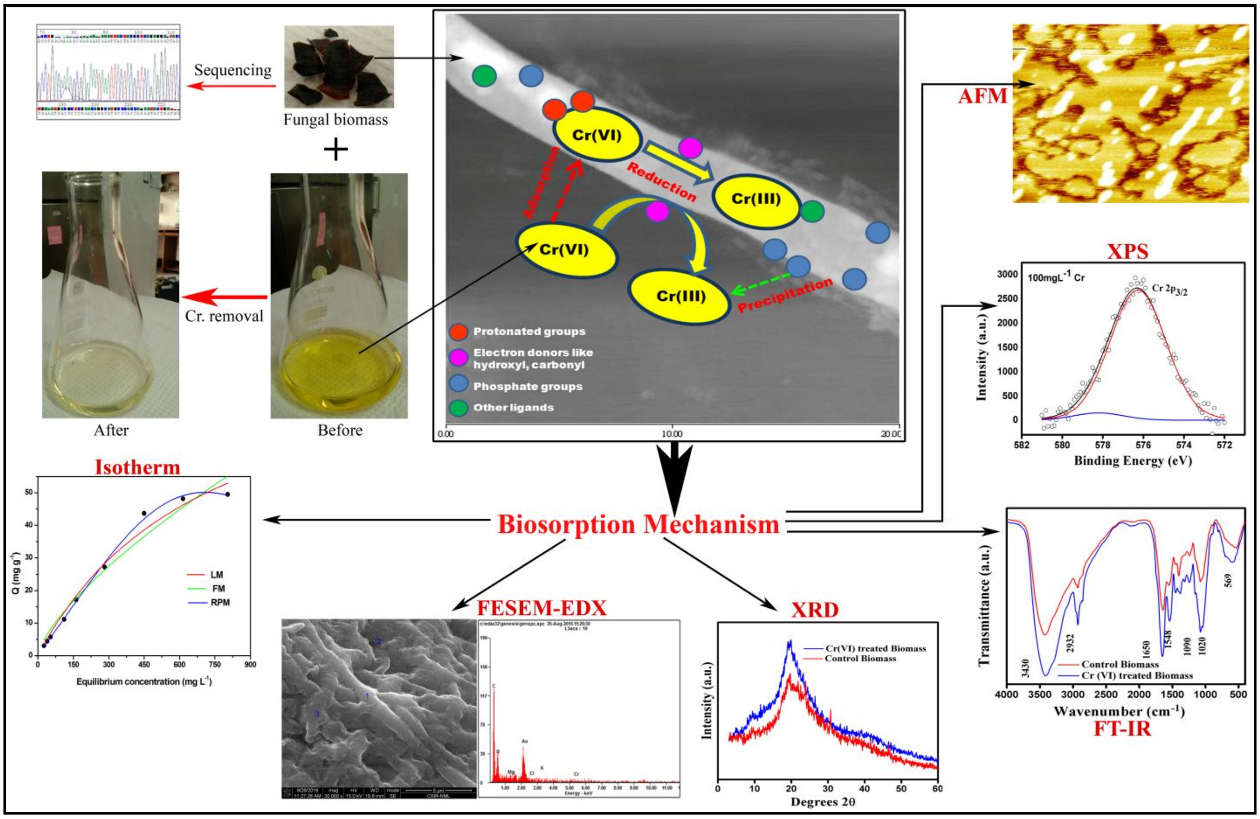

Methods

Isolation and molecular identification of the fungus. We isolated a novel fungus from the growing mat of another mushroom *Termitomyces clypeatus* MTCC-5091. The isolated fungus was routinely cultured in YPG media. After appropriate growth in the culture flasks, the fungal pellets were harvested through centrifugation (6000 rpm for 30 min) and washed with deionized water until pH of the water reached to 6.8-7.0 approximately.

For molecular identification, the fungal genomic DNA was isolated using the GeneJET genomic DNA purification kit according to the manufacturer protocol and the 18s rRNITS region was amplified by gradient PCR using customized primers [ITS1-‘TCCGTAGGTGAACCTGCGG’ and ITS4-‘TCCTCCGCTTATTGATATG’]. PCR (per 50 µl reaction mixture) conditions has been shown in tabulated form (Table S6a,b).Post PCR, the product was purified using QIAquick PCR purification kit following manufacturer protocol. The sequenced regions were submitted to the GenBank under the accession number KY007521.1. For quality checking purposes, approximately 125 ng of genomic DNA and 200 ng of purified PCR samples were separated by standard 1% agarose gel along with 1 kb DNA ladder (GeneRuler^{TM} 1 kb DNA ladder, thermo fisher scientific). After electrophoresis, the gel was visualized with ethidium bromide staining and photographed accordingly.

Equilibrium adsorption isotherm. The adsorption study of chromium on heat dried fungal biomass was carried out by batch equilibrium experiments. In brief, a known mass (~0.2 g) of adsorbent was suspended in 25 mL Cr (VI) solutions of different concentration (50-1200 mg L^{-1}) in a stoppered 250 mL flask and kept under isothermal condition (30°C) for 8 h at pH 3.0 in a shaking incubator (EYELA, Tokyo Rikakikai, Japan). We set up an individual flask for obtaining each of the data and therefore no correction was needed due to pipeting out of sampling volume.

The concentration of hexavalent chromium in the solution was determined spectrophotometrically at 540 nm (Thermo Scientific-Multiskan Go) after an equilibrium time. The amount of Cr (VI) adsorbed Q_e (mg g^{-1}), calculated by equation (2).

$$Q_e = V(C_i - C_e)/M \qquad (2)$$

Adsorption kinetic study. Kinetic data were obtained at designated time points (0-360 min) at initial chromium concentration of 100 mg L^{-1} with biomass dose of 8 g L^{-1}. At different time intervals, the solution was separated from the adsorbent material and the concentration of Cr (VI) in the solution was analyzed. The amount of dye adsorbed, Q_t (mg g^{-1}) at time t on the adsorbent, was calculated by equation (2). The linear equations for all the tested kinetic models and the methods of calculating the model parameters are presented in Table S7.

Batch biosorption experiments using Response Surface Methodology. Another batch experiments, designed through RSM, were conducted as described earlier.[1-4] In brief, experiments were conducted in 250 mL Erlenmeyer flasks containing 25 mL $K_2Cr_2O_7$ (100 mg L^{-1}) solution and appropriate amount of dried AMB by shaking (150 rpm) at 30°C. All the experimental data was performed and analyzed using Design Expert software (Version 10.0, stat-Ease, Inc., Minneapolis, United States). Software was used for fitting the equations that is developed along with regression and graphical analysis for evaluating the statistical significance. The fittest and the accuracy of the model were evaluated by F-value and the regression coefficient (R^2). The optimal condition for hexavalent chromium (Cr^{+6}) uptake by the fungal strain was obtained by solving the regression equations and the 3D responses for the variable parameters using Design-Expert software version 10.0. The mathematical model obtained using RSM was validated by conducting experiments on given optimal conditions. The differences in Cr(VI) concentration before and after biosorption were used to find out the efficiency (R) of

metal removal [percentage of hexavalent chromium adsorbed] by the biomass as equation (1).The amount of adsorbed Cr (VI) per gram biomass was obtained using equation (2).

$$Efficiency\ (R) = \frac{C_i - C_e}{C_i} \times 100 \qquad (1)$$

For each study, the control flask (without biomass) was also maintained. At the end of each experiment solutions were separated from the biomass by filtration through filter paper (Whatman no. 1), for analysis of Cr(VI) ions left in solution after biosorption. Care was taken to wash all glassware used for experimental purpose with extran (phosphate free) 50% (v v^{-1}), followed by 60% (v v^{-1}) nitric acid and subsequent rinsing with double-deionized water to remove any possible interference by other metals.

Determination of ΔG^o, ΔH^o and ΔS^oduringchromium adsorption. To investigate the thermodynamic nature of our biosorbent for Cr^{+6} biosorption, different parameters like ΔG° (Gibbs free energy), ΔH° (enthalpy) and ΔS° (entrophy) were calculated using the equations. The biomass dose was kept constant to 8 g L^{-1} in 100 mg L^{-1} concentration of $K_2Cr_2O_7$ solution. The thermodynamic parameters; change in Gibb's free energy (ΔG°), enthalpy (ΔH°), and entropy (ΔS°) of the adsorption process of hexavalent chromium onto oven dried AMB were calculated using Vant Hoff's equations (Table S7). The values of ΔH° and ΔS° were achieved from the slope and intercept of the plot ln K_c vs. 1/T, respectively.

Desorption and regeneration experiments. We carried out the regeneration experiments to ensure whether the fungal biomass has reusability property for commercial application. We used 0.1N NaOH and 0.1N HCl solutions as trial eluants for desorption studies. Chromium laden biomass was obtained by incubating the dried biomass into 100 mg L^{-1} potassium dichromate solution at time under experimental conditions. After the initial biosorption experiment, the biomass was placed in desorption solutions [0.1(N) NaOH, 0.1(N) HCl] for 24 h at 30°C under

shaking condition. The final Cr^{+6} concentrations were determined as described previously. After each cycle, the biosorbent was washed with double-deionized water and process repeated over successive cycles. The desorption ratio of Cr(VI) ions from biosorbent was calculated from the amount of Cr(VI) ions adsorbed onto biomass with the final Cr(VI) ion concentration in the desorption medium.[2] Desorption ratio was calculated from the following equation:

$$Desorption\ \ capacity : \frac{Cr(VI)\ desorbed\ \ in\ medium}{Cr(VI)\ ions\ \ adsorbed\ \ onto\ biosorbent} \times 100$$

Statistical analysis. All experimental results were expressed as mean data from triplicate sets, considering the *p* value is less than 0.05 using Design-Expert 10.0. The magnitude of regression coefficient and Chi-square were obtained using Origin 8.0 program.

References

1. Abigail, M., Samuel, M. S. & Chidambaram, R. Isotherm modelling, kinetic study and optimization of batch parameters using response surface methodology for effective removal of Cr(VI) using fungal biomass. *PloS ONE.* **10**, e0116884; 10.1371/journal.pone.0116884 (2015).

2. Samuel, M. S., Abigail, M. & Chidambaram, R. Biosorption of Cr(VI) by *Ceratocystis paradoxa* MSR2 using isotherm modelling, kinetic study and optimization of batch parameters using response surface methodology. *PloS ONE.* **10**, e0118999; 10.1371/journal.pone.0118999 (2015).

3. Majumder, R., Banik, S. P., Ramrakhiani, L. & Khowala, S. Bioremediation by alkaline protease (AkP) from edible mushroom *Termitomyces clypeatus*: optimization approach based on statistical design and characterization for diverse applications. *J. Chem. Technol. Biotechnol.* **90**, 1886-1896 (2015).

4. Hu, X., Wang, H. & Liu, Y. Statistical Analysis of Main and Interaction Effects on Cu(II) and Cr(VI) decontamination by nitrogen-doped magnetic graphene oxide. *Sci. Rep.* **6**, 34378; 10.1038/srep34378 (2016).

Supplementary Tables

Table S1: Box-Behnken (RSM) experimental design of three independent variables with actual and predicted responses

	Factor 1	Factor 2	Factor 3	Response (% chromium biosorption)	
Run Order	**A**: Contact time (h)	**B**: Biomass wt (g)	**C**: pH	Actual	Predicted
1	16	0.4	4	58.4	59.17
2	20	0.2	3	67.01	69.65
3	24	0.2	4	51.9	51.13
4	16	0.2	4	47.81	47.29
5	20	0.3	4	60.9	60.37
6	20	0.4	3	70.8	72.16
7	20	0.3	4	59.17	60.37
8	16	0.3	5	24.02	25.90
9	20	0.2	5	26.86	25.50
10	20	0.3	4	60.6	60.37
11	20	0.4	5	29.4	26.75
12	24	0.4	4	42.5	43.02
13	20	0.3	4	60.48	60.37
14	24	0.3	5	10.47	12.60
15	20	0.3	4	60.7	60.37
16	16	0.3	3	65.67	63.54
17	24	0.3	3	66.4	64.52

Table S2a: ANOVA for Response Surface Quadratic model for chromium biosorption

**ANOVA for Response Surface Quadratic model**						
**Source**	**Sum of Squares**	**Degree of freedom**	**Mean Square**	**F-Value**	**p-value Prob > F**	
Model	5060.78	9	562.31	104.97	< 0.0001	*significant*
A-Contact time	*75.83*	*1*	*75.83*	*14.16*	*0.0071*	
B-Biomass wt	*7.09*	*1*	*7.09*	*1.32*	*0.2878*	
C-pH	*4010.50*	*1*	*4010.50*	*748.67*	*< 0.0001*	
AB	*99.90*	*1*	*99.90*	*18.65*	*0.0035*	
AC	*50.98*	*1*	*50.98*	*9.52*	*0.0177*	
BC	*0.40*	*1*	*0.40*	*0.074*	*0.7933*	
A^2	*307.53*	*1*	*307.53*	*57.41*	*0.0001*	
B^2	*11.76*	*1*	*11.76*	*2.20*	*0.1820*	
C^2	*436.67*	*1*	*436.67*	*81.52*	*< 0.0001*	
Residual	37.50	7	5.36			
Lack of Fit	*35.60*	*3*	*11.87*	*25.05*	*0.0047*	*significant*
Pure Error	*1.89*	*4*	*0.47*			
Core Total	5098.28	16				

Table S2b: Statistical analysis of RSM data for the removal of hexavalent chromium

Std. Dev.	2.31	R-Squared	0.9926
Mean	50.77	Adjusted R-Squared	0.9832
C.V. %	4.56	Pred R-Squared	0.8877
PRESS	572.61	Adequate Precision	33.553

Table S3a: The Langmuir, Freundlich, and Redlich-Peterson isotherm constants for adsorption of Chromium (VI) onto *A. malaysianum* biomass surface

Isotherm models	Parameters	Values
Langmuir	Q_m (mg g^{-1})	100.69
	K_l (L mg^{-1})	1.38×10^{-3}
	χ^2	7.34
	R^2	0.9828
Freundlich	K_F (L g^{-1})	0.4837
	n	1.412
	χ^2	15.53
	R^2	0.9636
Redlich-Peterson	K_{RP} (L g^{-1})	0.1036
	α (L mg^{-1})	4.35×10^{-10}
	β	3.166
	χ^2	1.066
	R^2	0.9987

Table S3b: The PFO, PSO kinetic, intra-particle diffusion and film diffusion constants for adsorption of Chromium (VI) onto *A. malaysianum* biomass surface

1st Order (PFO)			Pseudo 2nd Order (PSO)			Intra-particle diffusion			Film diffusion	
K_1	Q_e	R^2	K_2	Q_e	R^2	K_p	C	R^2	K_f	R^2
0.0499 min^{-1}	4.36 mg g^{-1}	0.9837	0.0072 g mg^{-1} min^{-1}	5.45 mg g^{-1}	0.9907	0.268 (mg g^{-1} $min^{1/2}$	1.034	0.7877	0.02	0.987

Table S4: Thermodynamic parameters for adsorption of Chromium (VI) onto *A. malaysianum* biomass at different temperatures

Temperature (K)	ΔG^o (KJ mol^{-1})	ΔH^o (KJ mol^{-1})	ΔS^o (J $mol^{-1}K^{-1}$)
303	-4.5±0.08		
318	-5.2± 0.05	23.47	60.0
333	-7.3±0.98		

Table S5: Summary of FTIR peaks and its assignments

FTIR peaks	Sample Name		Representative range (cm^{-1})	Peak Assignment
	Control biomass	Metal treated biomass		
1	3422	3424	3500-3000	Hydrogen-bonded O–H stretching and N–H stretching of amines
2	2958<br>2927	2924<br>2854	3000-2800	C–H stretching (methyl, methylene groups)
3	–	1742	1760-1670	C=O stretching in carboxyl groups or ester groups
4	–	1708		C=O of the carboxylic groups of amino acids
5	1642<br>1549	1635<br>1547	1650-1500	C–N stretching in –CO–NH–; amide –C=O stretching<br>N–H bending in amide II
6	1453	1460	1400-1200	C–H bending and C–O stretching
7	1404	1379	1400-1000	C–N stretching, in-plane O–H bending, sulfur and phosphorus compound
8	1239	1246	1400-1000	C–H stretching in amide III and C–O stretching
9	1077	1078	1200-1000	P–O–C/P–O–H, C–OH stretching or Chromium-phosphate compound
10	1029	1033		
11	531	576	700-400	Nitro compounds and disulfide groups

Table S6a: PCR reaction mixture

gDNA	dNTP mix (10 mM)	Fwd. and Rev. Primer	Taq buffer	Taq DNA polymerase	DMSO	$MgCl_2$	MB grade water
4 µl (25 ng $µl^{-1}$)	1 µl	17 pmol each	5 µl	2.5 U	1.5 µl	2.5 mM	34.5 µl

Table S6b: PCR condition (total 35 cycles)

Steps	Temperature	Time
Initial denaturation	94°C	5 min
Denaturation	94°C	30 sec
Annealing	46.9°C / 50.1°C / 55.3°C gradient	30 sec
Extension	94°C	1.5 min
Final extension	94°C	7 min

Table S7: The models and equations used for the adsorption of Cr (VI) by *Arthrinium malaysianum* (Accession number: KY007521.1)

	Models	Equations		Model parameters
Adsorption isotherm	Langmuir	Linear: $\frac{C_e}{Q_e} = \frac{1}{Q_m} C_e + \frac{1}{K_l Q_m}$	Non-linear: $Q_e = \frac{Q_m K_l C_e}{1 + K_l C_e}$	K_l is energy of adsorption (L mg^{-1}), Q_e=equilibrium capacity of Cr (VI) adsorbed onto biomass (mg g^{-1}), C_e=equilibrium concentration of Cr (VI) solution (mg L^{-1}), and Q_m=maximum adsorption capacity (mg g^{-1})
	Freundlich	Linear: $\ln(Q_e) = \frac{1}{{}^{n}F} \ln(C_e) + \ln K_F$	Non-linear: $Q_e = K_F C_e^{\frac{1}{nF}}$	K_F is Freundlich constants (mg g^{-1}) and n is adsorption intensity of the adsorbent.
	Redlich-Peterson	Non-linear: $Q_e = \frac{K_{RP} C_e}{1 + \alpha C_e^{\beta}}$		K_{RP} (L g^{-1}) and α (L mg^{-1}) are Redlich-Peterson constants, β is Redlich-Peterson isotherm exponent.
Adsorption kinetics	Lagergren-first order	$\ln(Q_e - Q_t) = \ln Q_e - k_1 t$		k_1 is first-order rate constant (min^{-1}), Q_t = adsorption (mg g^{-1}) at any time t (min).
	Pseudo second order	$\frac{t}{Q_t} = \frac{1}{k_2 Q_e^{2}} + \frac{t}{Q_e}$		k_2 is the second order rate constant (g mg^{-1} min^{-1}).
	Intra-particle diffusion (Weber-Morris)	$Q_t = k_p t^{1/2} + C$		k_p is the intra-particle diffusion rate constant (mg g^{-1} $min^{1/2}$).
	Film diffusion	$\ln(1 - F) = -K_f \times t$		$F=Q_t/Q_e$ and K_f is the constant.
Adsorption thermodynamics	Vant Hoff's equation	$K_c = Q_e / C_e$<br>$\Delta G^\circ = -RT \ln K_c = \Delta H^\circ - T\Delta S^\circ$		ΔG^o = Gibbs free energy change (kJ mol^{-1}), R = the gas constant (J mol^{-1} K^{-1}), T = temperature (K), K_c= the equilibrium constant, ΔH^o= enthalpy change (kJ mol^{-1}) and ΔS^o= entropy change (kJ mol^{-1} K^{-1}).

Table S8: Experimental ranges and levels in the experimental design

Factors	Range and Level		
	**-1**	**0**	**+1**
A: Contact Time (h)	16	20	24
B: Biomass dose (g L^{-1})	4	6	8
C: pH	3	4	5

Table S9: Adsorption of chromium from leather industry (CLC-Calcutta Leather Complex) effluent by *A. malaysianum* biomass

Heavy metal	CETP inlet area (effluent 1)	Maximum range in leather industrial waste water	West Bengal Pollution Control Board standard	Residual chromium (VI) concentration
Concentration (mg L^{-1})				
Total Chromium	2.41	30-120	1-2	1.68 (~30% removal at effluent pH)<br>0.72 (~70% removal at pH 3)

Supplementary Figure S1

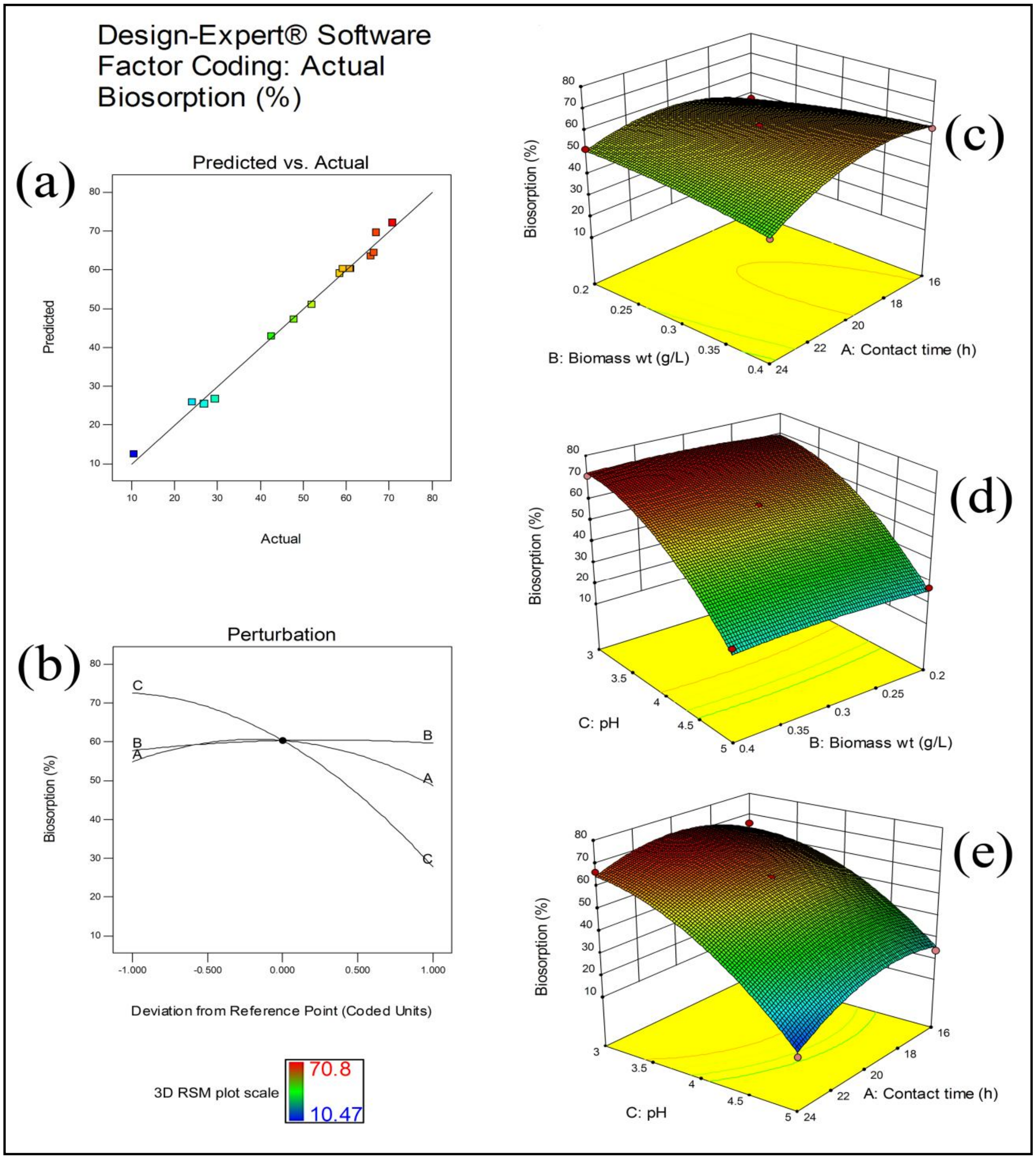

Figure S1: Response surface plots representing the percent biosorption of hexavalent chromium by *A. malaysianum* biomass. (a) Correlation plot between actual and predicted data of the RSM model based on the three factorial Box-Behnken design, (b) Overlay plot of perturbation of the three variables tested. Response surface 3D plots showing mutual interactions between (c) contact time-biomass dose, (d) biomass dose-pH and (e) contact time-pH on the removal (%) of Cr (VI).
